# Genome Sequence of *Gordonia* Phage Yvonnetastic

**DOI:** 10.1128/genomeA.00594-16

**Published:** 2016-07-07

**Authors:** Welkin H. Pope, Anshika Bandyopadhyay, Meghan L. Carlton, Meghan T. Kane, Niyati J. Panchal, Yvonne C. Pham, Zachary J. Reynolds, Michael S. Sapienza, Brian A. German, Jill E. McDonnell, Claire E. Schafer, Victor J. Yu, Emily C. Furbee, Sarah R. Grubb, Marcie H. Warner, Matthew T. Montgomery, Rebecca A. Garlena, Daniel A. Russell, Deborah Jacobs-Sera, Graham F. Hatfull

**Affiliations:** Department of Biological Sciences, University of Pittsburgh, Pittsburgh, Pennsylvania, USA

## Abstract

*Gordonia* bacteriophage Yvonnetastic was isolated from soil in Pittsburgh, PA, using *Gordonia terrae* 3612 as a host. Yvonnetastic has siphoviral morphology and a genome of 98,136 bp, with 198 predicted protein-coding genes and five tRNA genes. Yvonnetastic does not share substantial sequence similarity with other sequenced bacteriophage genomes.

## GENOME ANNOUNCEMENT

*Gordonia* spp. are implicated in foaming of wastewater in treatment plants (1–3) and are associated with opportunistic infections in hospital catheters (4, 5). Seventeen bacteriophages of *Gordonia* have been isolated, sequenced, and deposited in GenBank (6–9), and although these represent several different genome types, it is unclear whether the overall genomic diversity of the *Gordonia* phage population is similar to that reported for mycobacteriophages (10–15). The Science Education Alliance-Phage Hunters Advancing Genomics and Evolutionary Science (SEA-PHAGES) is a course-based research experience in which undergraduates perform authentic research through isolation and characterization of viruses using hosts of the phylum *Actinobacteria*, and it provides an opportunity for isolation and characterization of *Gordonia* phages (16).

Yvonnetastic was isolated from soil through direct plating of a filtered soil extract on lawns of *Gordonia terrae* 3612. Its morphology is siphoviral, with an isometric head and a tail of 400 nm in length. After purification and amplification, DNA was extracted and sequenced using an Illumina MiSeq platform with 140-bp single-end reads. Reads were assembled using Newbler into one major contig of 98,136 bp with discrete ends and 10-base single-stranded 3′ extensions with the sequence 5′-CGCGAAGCTC. Protein-coding genes were predicted using Glimmer (17), GeneMark (18), DNA Master, and Phamerator (19), and tRNAs were predicted using Aragorn version 1.3 (20); functional assignments were made using BLAST (21) and HHpred (22) against the publically available databases GenBank, the Protein Database, and pFamA. Yvonnetastic’s genome has 59.7% G+C content, somewhat lower than that of its host (67.8%), and it contains 198 predicted protein-coding genes and five tRNA genes. Functional assignments were made to 42 (21%) of the protein-coding genes. The majority of the Yvonnetastic genes are transcribed left to right, with the exception of genes in the leftmost 3 kbp and rightmost 10 kbp of the genome.

Yvonnetastic shows little similarity to currently sequenced phage genomes in its nucleotide sequence, and of the 198 predicted protein-coding gene products, <50% have amino acid similarity to genes encoded in ~1,500 sequenced phages of actinobacterial hosts. Those that do primarily match gene products encoded in other *Gordonia* phages and in mycobacteriophages. Among the putative predicted gene functions are the virion structure and assembly proteins, two glycosyltransferases, five HNH endonucleases, a RecET recombination system, an endonuclease VII, and an exonuclease VII.

The Yvonnetastic genome is unusual in that a lysis cassette including l-alanyl-d-glutamate peptidase, glycoside hydrolase, and holin genes is located amid the minor tail protein genes, and a putative lysin B gene is located near the right end of the genome, separated by >50 kbp from the other lysis genes. We also note that the putative integrase gene (*95*) is embedded within a long operon of closely linked rightwards-transcribed genes, and we have not been able to identify a putative *attP* site or predict a chromosomal *attB* site for phage integration. We have also been unable to identify putative immunity repressor gene, and the only predicted regulatory protein is the 67-residue gp109, which contains a predicted helix-turn-helix DNA binding motif.

### Nucleotide sequence accession number.

The Yvonnetastic genome is available from GenBank under the accession no. KU963248.
